# Exploring the Phylogeography of Ancient *Platycladus orientalis* in China by Specific-Locus Amplified Fragment Sequencing

**DOI:** 10.3390/ijms20163871

**Published:** 2019-08-08

**Authors:** Ermei Chang, Yuxin Tian, Caiyun Wang, Nan Deng, Zeping Jiang, Caixia Liu

**Affiliations:** 1 State Key Laboratory of Tree Genetics and Breeding, Key Laboratory of Tree Breeding and Cultivation of National Forestry and Grassland Administration, Research Institute of Forestry, Chinese Academy of Forestry, Beijing 100091, China; 2 Hunan Academy of Forestry, Changsha 410004, China; 3 Hunan Cili Forest Ecosystem State Research Station, Cili 427200, China; 4 Research Institute of Forest Ecology, Environment and Protection, Chinese Academy of Forestry, Beijing 100091, China

**Keywords:** *Platycladus orientalis*, ancient trees, population genomics, specific-locus amplified fragment sequencing

## Abstract

*Platycladus orientalis* (i.e., Chinese thuja) is famous for its lifespan spanning hundreds, and even thousands, of years. Most ancient *P. orientalis* populations are widely distributed in China, with accessible historical records, making them valuable genetic resources. In this study, the distribution pattern of ancient *P. orientalis* in China was analyzed based on 13 bioclimatic factors. Additionally, a specific-locus amplified fragment (SLAF) sequencing method was applied to detect single nucleotide polymorphisms (SNPs) in the genomes of 100 accessions from 13 populations. The resulting data revealed that the suitable areas for the distribution of ancient *P. orientalis* populations were accurately predicted with four main climatic factors. A total of 81,722 SNPs were identified from 461,867 SLAFs for 100 individuals, with an average sequencing depth of 10.11-fold and a Q30 value of 82.75%. The pair-wise genetic distance and genetic differentiation of 13 populations indicated that the BT-T population exhibited the largest divergence from the other populations. A neighbor-joining phylogenetic tree suggested the relationship between many individuals was inconsistent with the geographical location, possibly indicative of a history of transplantation and cultivation. All individuals were clustered into nine genotypes according to a structural analysis and the relationships between individuals were clarified in phylogenetic trees. This study highlights the importance of the de novo genome sequencing of ancient *P. orientalis* and may provide the basis for the conservation of *P. orientalis* genetic resources, the identification of supergene families, and the evaluation of related genetic resources.

## 1. Introduction

*Platycladus orientalis* (L.) Franco (common name: Chinese thuja) is an evergreen tree species of the family Cupressaceae that is endemic to China, with the common name of Chinese thuja. This species is one of the most widely used trees for landscaping in China, and it was planted in some temples, mausoleums, and gardens thousands of years ago. Additionally, *P. orientalis* is highly resistant to drought and cold conditions, as well as diseases, and is widely distributed throughout China, with the exception of Qinghai and Xinjiang provinces [1,2]. It is famous for its long lifespan of hundreds, or even thousands, of years [3]. One *P. orientalis* tree in the Yellow Emperor’s Mausoleum in Shanxi, China is believed to be more than 3000 years old. The mechanisms underlying plant longevity have attracted considerable attention from plant researchers interested in inhibiting senescence [4,5,6,7,8]. Most of the ancient *P. orientalis* populations distributed in China are well preserved with historical records, making them a valuable germplasm resource. Moreover, these trees represent historical relics that may be useful for revealing environmental, as well as social, changes. Regarding genetic diversity, these ancient trees are a genetic resource for wild and semi-wild ancestors. Additionally, the various genetic resources of ancient *P. orientalis* populations may be relevant as rare study materials for research aimed at delaying senescence.

Preserving the genetic diversity of ancient *P. orientalis* trees first requires that all ancient populations are protected. However, because of the limited funds available for this purpose as well as the push toward economic development at the expense of losing plant and natural resources, we must prioritize the protection of the most valuable groups. A precise understanding of population structures is critical for the delineation of species involved in conserving plant resources [9] as well as for determining the contemporary and historical barriers to gene flow [10,11]. Investigating population genetics is generally the main method for identifying high-priority populations for conservation. In other words, the populations with the highest genetic diversity, especially allelic diversity, should be prioritized.

Because of methodological limitations, a large proportion of population genetics studies have concentrated primarily on the dynamics of one, or a few, genetic loci [12]. All genes are integrated both by their physical proximity on chromosomes and various evolutionary processes [13,14]. Technological advances have made high-throughput sequencing a viable new strategy for studying population genetics and has enabled researchers to generate genome assemblies as well as high-density genetic marker datasets for most analyzed organisms [12,15,16,17]. High-density single nucleotide polymorphism (SNP) markers mapped to a reference genome can provide insights into processes that control the variations within genomes. Genomic markers can also be used to describe variations at the population and individual levels, even without positional information [18]. Specific-locus amplified fragment sequencing (SLAF-seq) is a strategy for de novo SNP discovery involving an enhanced reduced representation library sequencing method [19].

Ancient *P. orientalis* trees are an important genetic resource with genes related to longevity, stress resistance, and other desirable traits. In this study, we collected and investigated ancient *P. orientalis* materials and analyzed the distribution pattern of ancient *P. orientalis* in China based on 13 bioclimatic factors. Additionally, a SLAF-seq method was applied to reveal the genetic diversity of ancient *P. orientalis* populations. Consequently, high-quality SNPs evenly distributed among the *P. orientalis* chromosomes were obtained inexpensively. The oldest known *P. orientalis* tree (over 3000-years-old) worldwide (i.e., “Father of the World Cypress”) was included as a study sample. The results of this study provide a solid basis for the protection and characterization of ancient *P. orientalis* trees, and also provide important information regarding *P. orientalis* genetic resources for future related investigations and applications.

## 2. Results

### 2.1. Potential Tree Distribution and Effects of Climatic Factors

**Figure 1:**
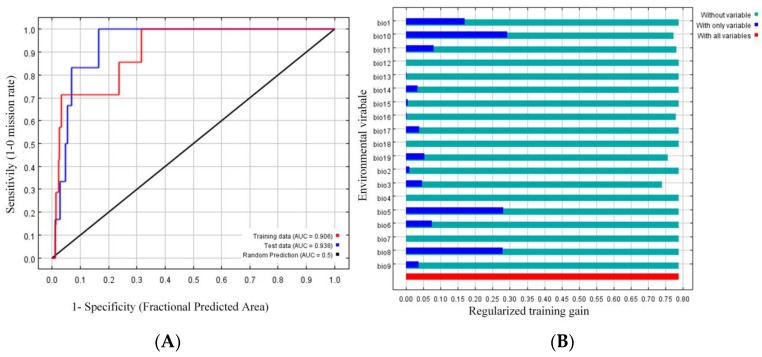
The receiver operating characteristic (ROC) curve (**A**) and jackknife test results (**B**) for the ancient *P. orientalis* distribution model. (**A**): The red and blue curves represent the training and test data, respectively, whereas the black line represents a random prediction; (**B**): Light blue bars correspond to no variables, dark blue bars correspond to only one variable, and red bars correspond to all variables.

**Figure 2:**
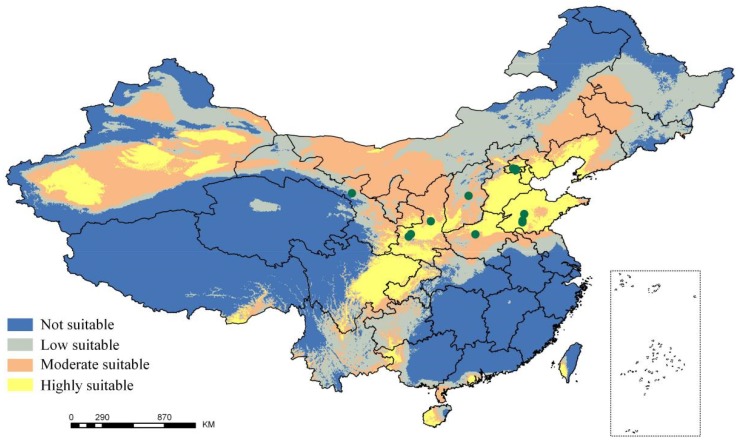
Predicted distribution of suitable regions for ancient *P. orientalis* populations. The green points represent the location of sample collection sites.

The maximum entropy model was used for predicting the areas in which the ancient *P. orientalis* populations in China are distributed. The ROC curve of the model is presented in Figure 1A. The AUC for the training data subset and the test data subset was close to 1 (0.906 and 0.938, respectively), which indicated the models can accurately distinguish the distribution of the ancient *P. orientalis*. On the basis of the jackknife test results (Figure 1B), four climatic factors were identified as the main influencing climatic factors (i.e., Bio1: Annual mean temperature, Bio5: Maximum temperature of the warmest month, Bio8: Mean temperature of the wettest quarter, and Bio10: Mean temperature of the warmest quarter). The simulated distribution of the ancient *P. orientalis* populations in China is presented in Figure 2. The highly and moderately suitable areas were mainly concentrated in Beijing, Hebei, East of Sichuan, West of Xinjiang, south of Shanxi, and Southeast of Hainan and Taiwan.

### 2.2. Statistical Analysis of the Sequencing Data

The distribution of sequence bases is presented in Supplementary Figure S1. The base distribution fluctuated depending on the restriction enzyme site and PCR, especially the first two bases. Digestion efficiency is a key index for evaluating the success of SLAF-seq. Our results suggested that 99.6% normal digestion corresponds to a highly efficient digestion. Additionally, more than 42.13 million reads for 100 individuals were generated in this study. As mentioned in the Materials and Methods section, a Q value of 30 indicated the error rate was 1/1000. The Q30 value (Q < 30) and GC content for the reads were 82.75% and 34.43%, respectively.

### 2.3. SLAF Tags and SNP Sites

In this study, a total of 42,299 high-quality SLAF tags were identified after a sequence alignment with the reference genome. The high-quality SLAFs were selected with a total depth threshold of 1000-fold, and the average sequencing depth was 10.11-fold. Of these SLAFs, 32,870 were identified as polymorphic, and 9249 were identified as nonpolymorphic (Table 1, Supplementary File S1). Finally, 81,722 SNPs with a minor allele frequency ≥0.05 and integrity ≥80% were derived from 461,867 SLAFs for the 100 accessions, with a heterozygosity ratio of 0.0198.

### 2.4. Genome-Wide Estimates of Genetic Diversity

The pair-wise genetic distance (Ds) and genetic differentiation (Fst) of 13 ancient *P. orientalis* populations were calculated (Table 2). The Ds calculated with Nei’s method ranged from 0.229 to 0.365, with an average of 0.288. The largest genetic distance (0.365) was associated with the Zhongshan Park (BJ-Z) and Weiwu Temple (GS-W) populations, followed by the BJ-Z and Jinci Temple (SX-J) populations. The smallest genetic distance (0.229) was calculated for the Cemetery of Confucius (SD-C) and Mengmu Cemetery (SD-M) populations. The Fst value ranged from 0.024 to 0.386, with an average of 0.147. The highest Fst value (0.386) was recorded for the BJ-Z and SX-J populations, followed by the Songyang Academy (HN-S) and SX-J populations. The lowest Fst value (0.024) was calculated for the Tiantan Park (BJ-T) and Wofuo Temple (BJ-W) populations. Moreover, the Ds and Fst values between BJ-Z and the other populations were highest and were greater than the average values. Additionally, the Pi and Tajima’s D value of all populations were calculated and list in Supplementary Files S2 and S3.

**Figure 3:**
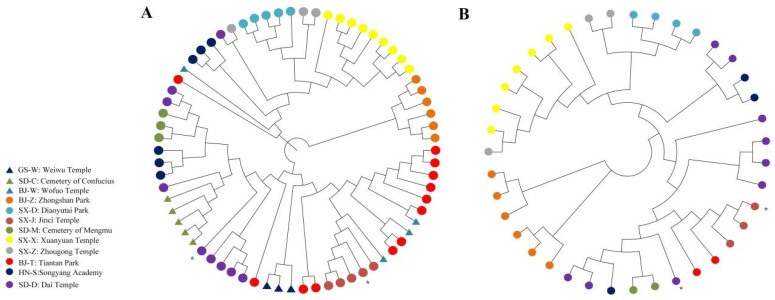
Phylogenetic tree constructed according to the neighbor-joining method with polymorphic SNPs. (**A**): Phylogenetic tree based on *P. orientalis* trees over 500 years old; (**B**): Phylogenetic tree based on *P. orientalis* trees over 1000 years old.

To ascertain the divergence and relationships among the populations, a phylogenetic tree consisting of 100 accessions was constructed based on the identified SNPs (Supplementary Figure S2). The BJ-Z individuals were grouped together and were separated from the other populations, whereas the Xuanyuan Temple (SX-X), SX-J, and SD-M individuals were also grouped together, but they were also clustered with other populations. The relationship between many individuals was inconsistent with the geographical location. For example, Ditan Park (BJ-D) individuals were grouped with individuals from various populations. This result may have been because the younger trees were the progeny of other older trees and some younger trees may have been transplanted from other areas. Therefore, phylogenetic trees for individuals over 500 and 1000 years old were constructed to resolve the relationships among older individuals. Accordingly, 70 and 39 individuals over 500 and 1000 years old, respectively, were identified, and the corresponding phylogenetic trees (Figure 3A) revealed that the over 500-year-old individuals in five populations [Diaoyutai Park (SX-D), SX-J, SD-C, GS-W, and BJ-Z] were grouped together. Additionally, the over 1000-year-old individuals in two populations (SX-D and BJ-Z) clustered together (Figure 3B).

**Figure 4:**
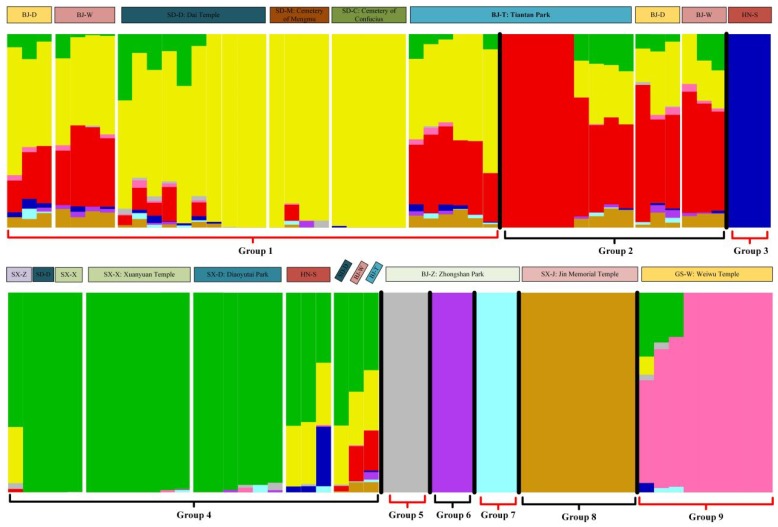
Genetic structure of ancient *P. orientalis*. The length of each colored segment represents the proportion of the individual’s genome from K = 9 ancestral populations.

On the basis of the SNP data, the Admixture software was used to analyze the genetic structure of the ancient *P. orientalis* populations. A total of 100 accessions were divided according to the cross-validation error rate (Supplementary Figure S3A). Additionally, K was initially set as 1–30 prior to clustering (Supplementary Figure S3B). The peak value of ΔK was used to determine the number of groups. According to the results, when K = 9, nine separate groups were identified, with each group including 3–32 members. The length of each colored segment in Figure 4 represents the proportion of an individual’s genome that was derived from nine ancestral populations. Groups 8 and 9 consisted of the individuals from the GS-W and SX-J locations, respectively, whereas Groups 5, 6, and 7 comprised the individuals from BJ-Z. Group 3 included the HN-S population. Group 2 consisted of all individuals from Zhougong Park (SX-Z), SX-X, and SX-D as well as four individuals from BJ-W, BJ-T, and Dai Temple (SD-D). Group 2 also included some of the individuals from BJ-D, BJ-W, and BJ-T. Group 1 was the largest group, with individuals from six populations.

## 3. Discussion

In this study, the 100 ancient *P. orientalis* accessions collected from 13 sites represented the prevailing ancient *P. orientalis* populations in China. All trees were well protected and had available historical records, thereby ensuring the accuracy of the analysis. The distribution of these samples was used to predict the potential distribution in other suitable areas. A model accuracy test confirmed that the simulation results were of a high standard, implying the developed model was appropriate for the objectives of this study. On the basis of the generated data, we predicted the regions in China potentially suitable for ancient *P. orientalis* populations. In this prediction, Bio1 (Annual mean temperature), Bio5 (Maximum temperature of the warmest month), Bio8 (Mean temperature of the wettest quarter), and Bio10 (Mean temperature of the warmest quarter) were considered the climate factors with the strongest influences. In fact, the main disease causing the death of *P. orientalis* trees is leaf blight, which is caused by biotic factors. This disease occurs at elevated temperatures, especially during the warmest quarter (6-9), which is also the wettest quarter. In this quarter, the temperature and humidity can promote the propagation and spread of pathogenic organisms, with *Semanotus bifasciatus* Motschulsky and *Pityogenes* spp. L. infecting trees more easily than normal. These infections often accelerate the death of *P. orientalis* populations. Consequently, temperature is an important factor affecting the distribution and reproduction of *P. orientalis*, especially under humid conditions. This has been confirmed by our predictions, which may be relevant for identifying the factors influencing the predicted appropriate distribution. Thus, our findings provide the basis for future investigations regarding the protection of ancient *P. orientalis*.

Advances in high-throughput next-generation sequencing have allowed researchers to generate genomic marker datasets for particular species. These datasets can be applied to address a broad range of questions related to population genetics and evolution more precisely than with traditional methods. In this study, 100 accessions were analyzed with a SLAF-seq method, with an average sequencing depth of 10.11-fold that confirmed the accuracy of the analysis [15,20]. The Q30 value of 82.75% verified the reliability of our results. A total of 81,722 SNPs were identified from the 461,867 SLAFs for 100 individuals, which is consistent with the results of a SLAF-seq investigation of soybean [15]. In a previous study of a *P. orientalis* population involving a more traditional method, 1,680 polymorphic bands were identified based on AFLPs [21]. Additionally, 34 polymorphic sites were identified in a *Thuja sutchuenensis* Franch. population based on a RAPD method [22]. The number of SNPs identified in the current SLAF-seq study, with a high genome coverage, far exceeded the number of polymorphisms detected using traditional methods.

According to our phylogenetic analysis, all of the individuals from the BJ-Z population clustered together without other individuals (Supplementary Figure S2), suggesting the origin of the BJ-Z population differs from that of the other populations; the pair-wise Ds and Fst values support this suggestion. The genetic structural analysis revealed that the BJ-Z population comprises three genotypes, which is in accordance with the phylogenetic results. Some populations (e.g., HN-S and BJ-T) consisted of two or more genotypes, indicative of a relatively high gene flow.

To accurately clarify the selection between populations, genome-wide variations were measured based on the SNPs between the BJ-Z and SD-D populations. The θπ ratios and Fst values were calculated with a sliding window analysis (100 kb windows and 10 kb steps). The threshold values for selecting genomic regions was set as the 5% left and right tails of the empirical θπ ratio distribution (the θπ ratios were 0.24 and 3.03). Regions above the horizontal dashed line (the 5% right tail of the empirical Fst distribution, where Fst is 0.361) were identified as appropriate regions for BJ-Z (blue points) and GS-W (green points) populations (Supplementary Figure S4; Supplementary File S4).

This study highlights the value of the de novo sequencing of ancient *P. orientalis* genomes to elucidate the genomic patterns in a phylogenetic framework. The SLAF-seq analysis of various ancient *P. orientalis* populations revealed the genetic diversity and the geographically distinct demographic histories of the trees, which were consistent with the results of phylogenomic analyses. The data presented herein may represent the basis for future studies related to the conservation of *P. orientalis* genetic resources, the identification of supergene families, and the evaluation of related genetic resources.

## 4. Materials and Methods

### 4.1. Sample Preparation

A total of 100 ancient *P. orientalis* accessions were included in the present study to represent the genetic and geographical diversity in China. The sample collection sites ranged from 34°N to 40°N and from 102°E to 117°E. All samples were collected from historical cultural monuments in China, including temples, mausoleums, and royal gardens that have existed for thousands of years (Table 3). For the purpose of this study, *P. orientalis* trees that were over 100 years old were considered as ancient trees. Tree age was based on the diameter at breast height (DBH) of selected trees as well as historical records available from local government departments responsible for managing ancient trees. All individuals included in this study were over 100 years old, including 30 individuals 100 to 500 and 500 to 1000 years old as well as 40 individuals 1000 to 3000 years old. The DBH of these trees exceeded 80 cm, and most of the trees were planted for greening and celebrations. The DBH of the oldest *P. orientalis* tree (approximately 3000 years old) was 7800 cm. New leaves were collected from the south-facing middle canopy layer of pest- and disease-free trees exposed to similar daily illumination times. Samples were flash frozen in liquid nitrogen.

### 4.2. Potential Distribution Predictions

A maximum entropy model was used to predict the potential distribution of ancient *P. orientalis*. This investigation involved a total of 13 sites. Environmental data were derived from the WorldClim database (http://www.worldclim.org/). Additionally, 19 bioclimatic variables were considered in this research (Table 4), with a layer resolution of 30″. An administrative map of China (1:400 million) was obtained from the National Geomatics Center of China. The maximum entropy model was constructed with the Maxent (version 3.4.1) program, and 75% of the samples were used to establish a training subset, whereas the remaining samples were used to verify the reliability of the model.

The receiver operating characteristic (ROC) curve was drawn after the maximum entropy model was constructed, and the area under the curve (AUC) was used to test the accuracy of the model. The evaluation criteria for the AUC were as follows [23,24]: Failed (0.5–0.6), poor (0.6–0.7), common (0.7–0.8), good (0.8–0.9), and great (0.9–1.0). Additionally, the jackknife method was used to identify the climatic factors with the biggest influence [25]. Finally, the ancient *P. orientalis* distribution areas were divided into the following four grades according to the natural discontinuity method: Not suitable, low suitability, moderate suitability, and highly suitable.

### 4.3. SLAF Library Construction and Sequencing

For each accession, the CTAB method was used to isolate DNA from the dried leaves of a single plant for a subsequent SLAF-seq analysis [19]. Because the *P. orientalis* genome has not been sequenced, the *Picea glauca* genome was selected as the reference sequence for predicting restriction enzyme sites (genome size: 24.63 Gb; GC content: 38.51%) [26] based on a previous study [27]. The restriction enzymes and sizes of the digested fragments were evaluated according to the following criteria: 1) SLAF tags per genome >10,000; 2) even distribution in unique genomic regions; and 3) repeated SLAFs avoided as much as possible. Digested genomic fragments with differing lengths were simulated in silico, after which restriction enzymes were selected based on the uniqueness and uniformity of the alignment of the simulated fragments to the reference genome. Accordingly, two restriction enzymes (*Eco*RV and *Sca*I) were selected, and a 464 to 494 bp fragment was identified as a SLAF tag. A total of 55,421 SLAF tags were predicted. For each sample, 10 μg genomic DNA (≥100 ng μl^−1^) was digested and incubated at 37 °C with *Eco*RV [New England Biolabs (NEB), Ipswich, MA, USA], T4 DNA ligase (NEB), ATP (NEB), and an *Sca*I adapter. The restriction-ligation reactions were heat-inactivated at 65 °C, and the samples were digested with *Sca*I at 37 °C. The restriction-ligation samples (diluted) were used as the template for a PCR amplification with Taq DNA polymerase (NEB), dNTPs, and an *Eco*RV-primer containing a barcode. The E.Z.N.A.^®^ Cycle Pure Kit (Omega Bio-Tek, Norcross, GA, USA) was used to purify the PCR products. Pooled samples were incubated at 37 °C with *Eco*RV, T4 DNA ligase, ATP, and a Solexa^™^ adapter (Illumina Co., San Diego, CA, USA), after which they were purified with a Quick Spin column (Qiagen, Hilden, Germany) and separated on a 2% (w/v) agarose gel. Fragments that were 400–500 bp were isolated with the Gel Extraction Kit (Qiagen). These fragments were then used as the template for a PCR amplification with the Phusion^™^ Master Mix (NEB) and the Solexa Amplification primer mix to add the barcode. The amplified fragments that were 450–500 bp long were excised and purified as previously described and diluted for the paired-end sequencing, which was completed with the Genome Analyzer II high-throughput sequencing platform (Illumina, Inc., San Diego, CA, USA).

### 4.4. Sequencing Data Processing and SNP Calling

The sequence error rate was estimated using rice (*Oryza sativa* indica) data as a control to prevent false positive results [28]. The Q value was used to evaluate the single-base error rate of high-throughput sequencing [Q = −10 × log10e (e represents the single-base error rate)]. The base distribution of SLAF-seq results was also examined according to the restriction sites and PCR. The reference sequence was selected based on the sequencing depth of each SLAF tag, and all reads were aligned to the reference with the Burrows Wheeler Aligner [29]. The Genome Analysis Toolkit [30] and SAMtools [31] were used for SNP calling, and the resulting data were combined to form the SNP dataset. The SNPs with a minor allele frequency <5% and integrity <80% were excluded from the dataset.

### 4.5. Phylogenetic Analysis

Phylogenetic relationships were deduced from the SLAF-seq data. The SNPs were used to construct phylogenetic trees for all accessions according to the neighbor-joining method [32] in the MEGA5 program [33]. The genetic structures were analyzed with the Admixture software [34]. A principal component analysis was performed based on the SNP differences in individual genomes to divide the accessions into subpopulations with R (3.4.4). To analyze the organization of the populations based on multilocus genotype information, the genetic population structure was examined with the Admixture software [34]. To clarify the evolutionary process, K (genetic clusters) was predefined as 1–30 before clustering. The cross-validation error rate (ΔK) was used to determine K (minimum value). We also imported the same set of SNPs into the GenoDive program [35] to perform various population differentiation tests.

## 5. Conclusions

In summary, we herein reveal the genetic diversity of ancient *P. orientalis* at the population and species level. Considerable genetic differentiation was observed among populations. A phylogenetic tree suggested the relationship between many individuals was inconsistent with the geographical location, possibly indicative of a history of transplantation and cultivation. All individuals were clustered into nine genotypes according to a structural analysis. On the basis of these findings, strategies have been proposed for conserving ancient *P. orientalis* populations.

## Figures and Tables

**Table 1:** Summary statistics for all populations of specific-locus amplified fragment sequencing (SLAF-seq) data processing.

Population	Sample Number	Total Reads	Q30 (%)	GC Content (%)	SLAF Number	Average Depth	Polymorphic SLAF	SNP Number	Heterozygosity (%)
SD-D	12	5,851,890	82.63	34.47	352,123	11.44	314,150	3,069,621	0.43
BJ-T	16	7,163,133	82.56	34.39	456,407	10.84	406,430	3,912,571	0.36
SD-M	4	1,893,816	82.62	34.50	118,467	10.83	105,589	1,036,869	0.36
BJ-W	8	3,058,893	82.65	34.54	231,879	8.80	206,623	1,963,840	0.35
HN-S	6	2,307,247	82.66	34.49	170,807	9.36	152,372	1,426,753	0.35
SX-D	6	2,514,465	82.97	34.23	164,998	11.02	147,496	1,358,212	0.38
GS-W	9	3,300,962	83.00	34.53	247,813	9.28	220,406	2,015,626	0.28
SX-Z	2	57,506	83.25	34.25	57,506	19.34	51,351	485,378	0.68
SD-C	5	201,0243	82.42	34.31	145,908	9.67	130,346	1,231,667	0.34
BJ-D	6	2,356,156	82.81	34.38	175,280	9.37	156,352	1,478,408	0.35
BJ-Z	9	3,885,951	82.80	34.25	256,074	10.45	228,777	2,170,506	0.24
SX-J	8	3,270,113	82.85	34.21	236,456	9.85	209,765	1,974,304	0.34
SX-X	9	3,676,621	83.10	34.78	252,694	9.37	224,749	2,093,102	0.20

**Table 2:** Estimates of evolutionary divergence (Ds) (down) and genetic differentiation (Fst) (up) over sequence pairs and between groups.

Population	SX-D	BJ-D	GS-W	SX-J	SD-C	SD-M	SX-X	SD-D	BJ-T	BJ-W	HN-S	BJ-Z	SX-Z
SX-D		0.302	0.330	0.315	0.312	0.309	0.261	0.296	0.302	0.306	0.309	0.338	0.261
BJ-D	0.109		0.286	0.237	0.253	0.254	0.291	0.254	0.237	0.244	0.266	0.330	0.298
GS-W	0.163	0.125		0.287	0.294	0.291	0.323	0.294	0.285	0.294	0.307	0.365	0.324
SX-J	0.334	0.265	0.353		0.262	0.265	0.300	0.262	0.234	0.235	0.277	0.345	0.306
SD-C	0.144	0.076	0.160	0.314		0.229	0.302	0.241	0.255	0.264	0.259	0.330	0.310
SD-M	0.143	0.078	0.155	0.317	0.074		0.297	0.245	0.258	0.264	0.262	0.325	0.298
SX-X	0.067	0.091	0.150	0.318	0.127	0.124		0.286	0.291	0.294	0.299	0.337	0.257
SD-D	0.095	0.041	0.121	0.278	0.051	0.058	0.077		0.258	0.263	0.262	0.328	0.290
BJ-T	0.107	0.026	0.122	0.259	0.076	0.081	0.089	0.044		0.242	0.272	0.336	0.301
BJ-W	0.105	0.028	0.123	0.254	0.079	0.080	0.084	0.042	0.024		0.279	0.340	0.301
HN-S	0.166	0.117	0.197	0.354	0.138	0.142	0.151	0.105	0.122	0.123		0.339	0.298
BJ-Z	0.171	0.157	0.222	0.386	0.182	0.180	0.163	0.146	0.159	0.157	0.217		0.336
SX-Z	0.122	0.100	0.071	0.328	0.135	0.134	0.108	0.094	0.097	0.098	0.164	0.190	

**Table 3:** Locations of ancient *Platycladus orientalis* samples.

Population Code	Location	Number of Individuals	Latitude (N)	Longitude (E)	Average Age (Years)
BJ-T	Tiantan Park, Beijing Municipality	16	39°52	116°24′	650
BJ-D	Ditan Park, Beijing Municipality	6	39°57′	116°24′	191.7
BJ-W	Wofuo Temple, Beijing Municipality	8	40°0′	116°12′	437.5
BJ-Z	Zhongshan Park, Beijing Municipality	9	39°54′	116°23′	1000
SD-M	Cemetery of Mengmu, Qufu City, Shandong Province	4	35°29	116°58′	800
SD-D	Dai Temple, Tai’an City, Shandong Province	12	36°11′	117°07′	1341.7
SD-C	Cemetery of Confucius, Qufu City, Shandong Province	5	35°37′	116°59′	500
HN-S	Songyang Academy, Zhengzhou City, Henan Province	6	34°28′	113°01′	1250
SX-Z	Zhougong Temple, Baoji City, Shaanxi Province	2	34°29′	107°36′	1300
SX-D	Diaoyutai Park, Baoji City, Shanxi Province	6	34°16	107°25′	991.7
SX-J	Jinci Temple, Taiyuan City, Shanxi Province	8	37°42′	112°26′	912.5
SX-X	Xuanyuan Temple, Yan’an City, Shanxi Province	9	35°35′	109°16′	1744.4
GS-W	Weiwu Temple, Wuwei City, Gansu Province	9	37°55′	102°38′	381.1

**Table 4:** 19 bioclimatic variables used to build the model.

Code	Description
Bio1	Annual Mean Temperature
Bio2	Mean Diurnal Range (Mean of monthly (max.temp.–min.temp.))
Bio3	Isothermality (BIO2/BIO7) (* 100)
Bio4	Temperature Seasonality (standard deviation *100)
Bio5	Max Temperature of Warmest Month
Bio6	Min Temperature of Coldest Month
Bio7	Temperature Annual Range (BIO5–BIO6)
Bio8	Mean Temperature of Wettest Quarter
Bio9	Mean Temperature of Driest Quarter
Bio10	Mean Temperature of Warmest Quarter
Bio11	Mean Temperature of Coldest Quarter
Bio12	Annual Precipitation
Bio13	Precipitation of Wettest Month
Bio14	Precipitation of Driest Month
Bio15	Precipitation Seasonality (Coefficient of Variation)
Bio16	Precipitation of Wettest Quarter
Bio17	Precipitation of Driest Quarter
Bio18	Precipitation of Warmest Quarter
Bio19	Precipitation of Coldest Quarter

## Supplementary Materials

Supplementary materials can be found at https://www.mdpi.com/1422-0067/20/16/3871/s1. Supplementary Figure S1: Sequence base distribution of ancient *P. orientalis*. Supplementary Figure S2: Phylogenetic tree comprising 100 accessions constructed according to the neighbor-joining method with polymorphic SNPs. Supplementary Figure S3: Population structure of 100 accessions (A) and the individual clusters corresponding to each K value as determined with the Admixture software (B). Supplementary Figure S4: Distribution of θπ ratios and Fst values and the regional distribution of population differentiation. Blue and green points represent the selected regions for the BJ-Z and GS-W populations. Supplementary File S1: Summary of the sequencing and SNP detection results for 100 accessions. Supplementary File S2: Pi value of all populations. Supplementary File S3: Tajima’s D value of all populations. Supplementary File S4: Selected regions for the GS-W and BJ-Z populations.
