# Supplementary material for: Exploring the Phylogeography of Ancient Platycladus orientalis in China by Specific-Locus Amplified Fragment Sequencing
Source: Int J Mol Sci. 2019 Aug 8;20(16):3871. doi: 10.3390/ijms20163871 (PMC6719015; doi:10.3390/ijms20163871)
Supplement: Supplementary file 1 [file ijms-20-03871-s001.zip › ijms-542953-supplementary-XML/supplementary figures S1-S4.pdf]

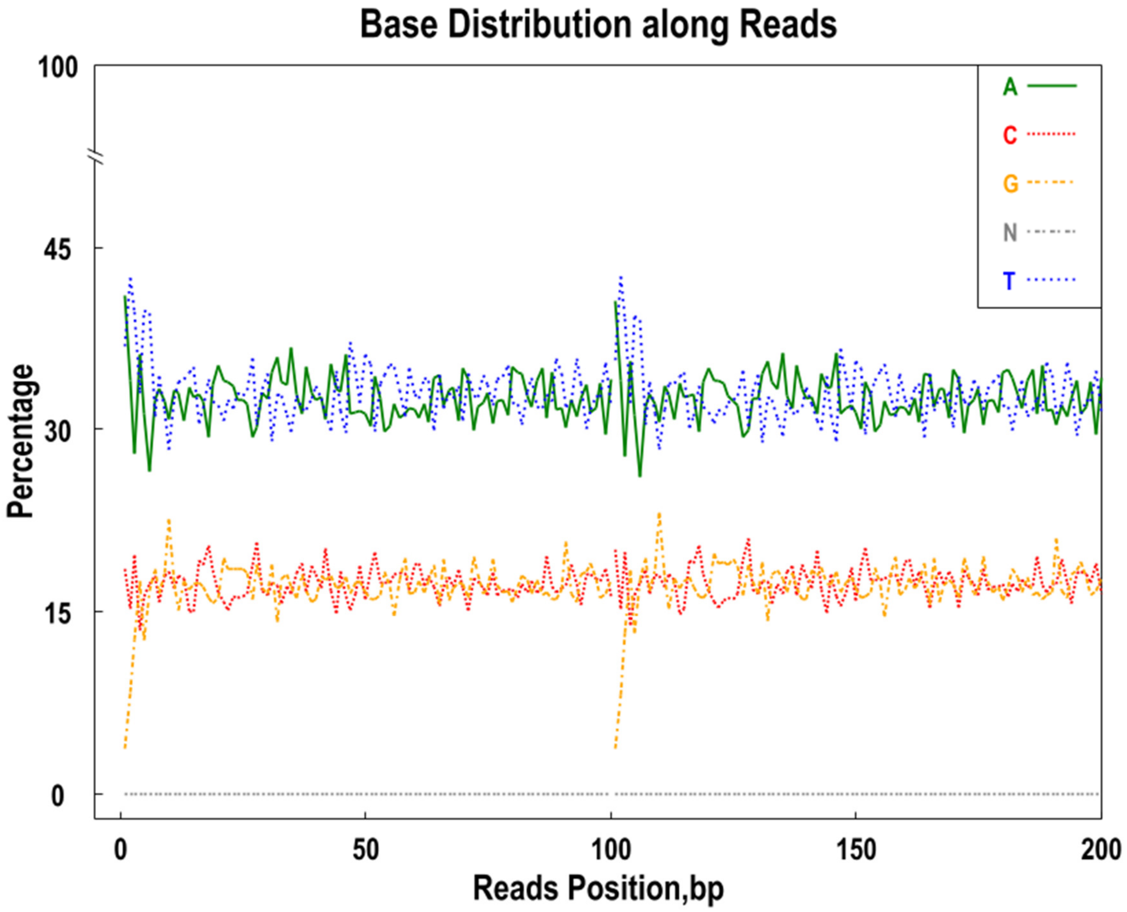

Supplementary Figure S1: Sequence base distribution of ancient *P. orientalis*.

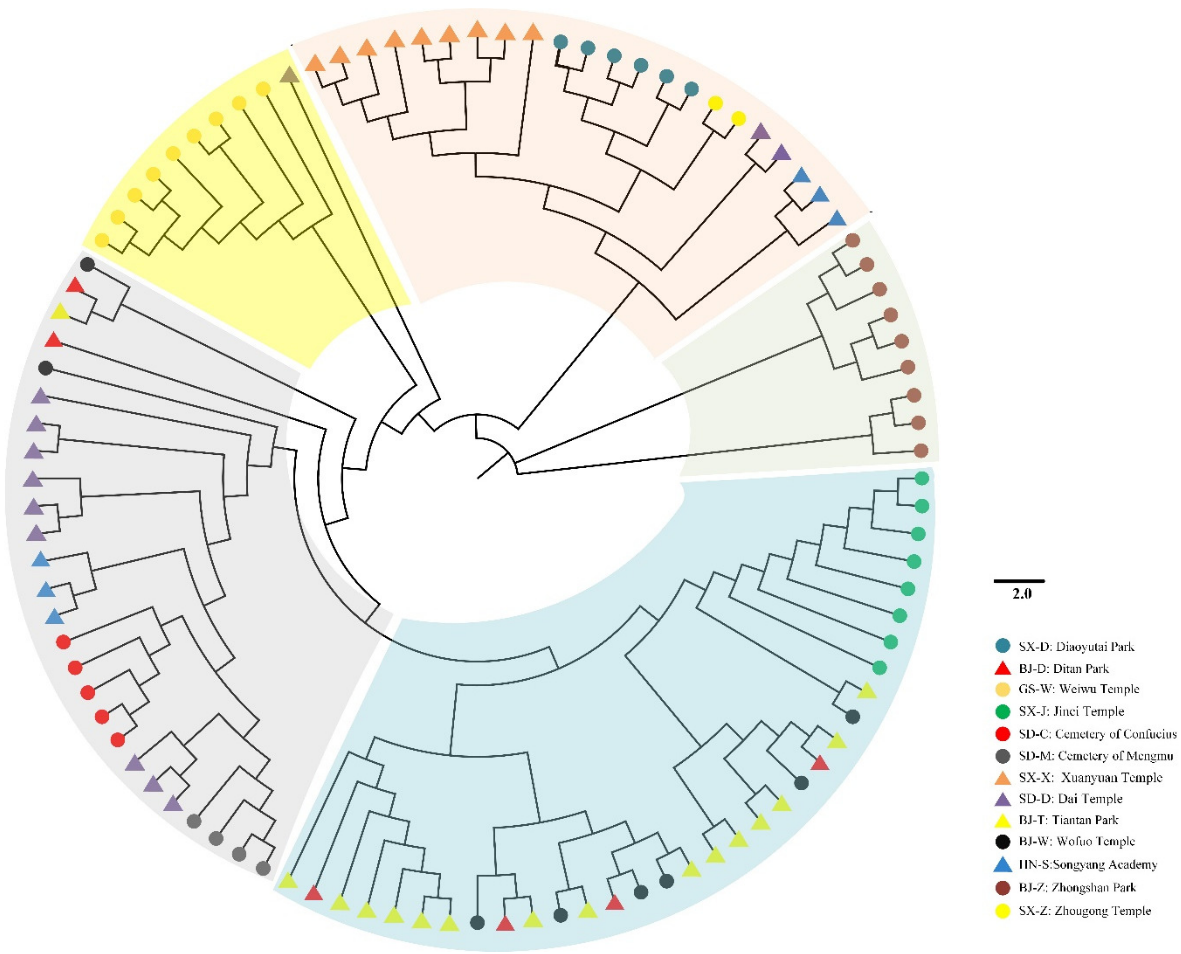

Supplementary Figure S2: Phylogenetic tree comprising 100 accessions constructed according to the neighbor-joining method with polymorphic SNPs.

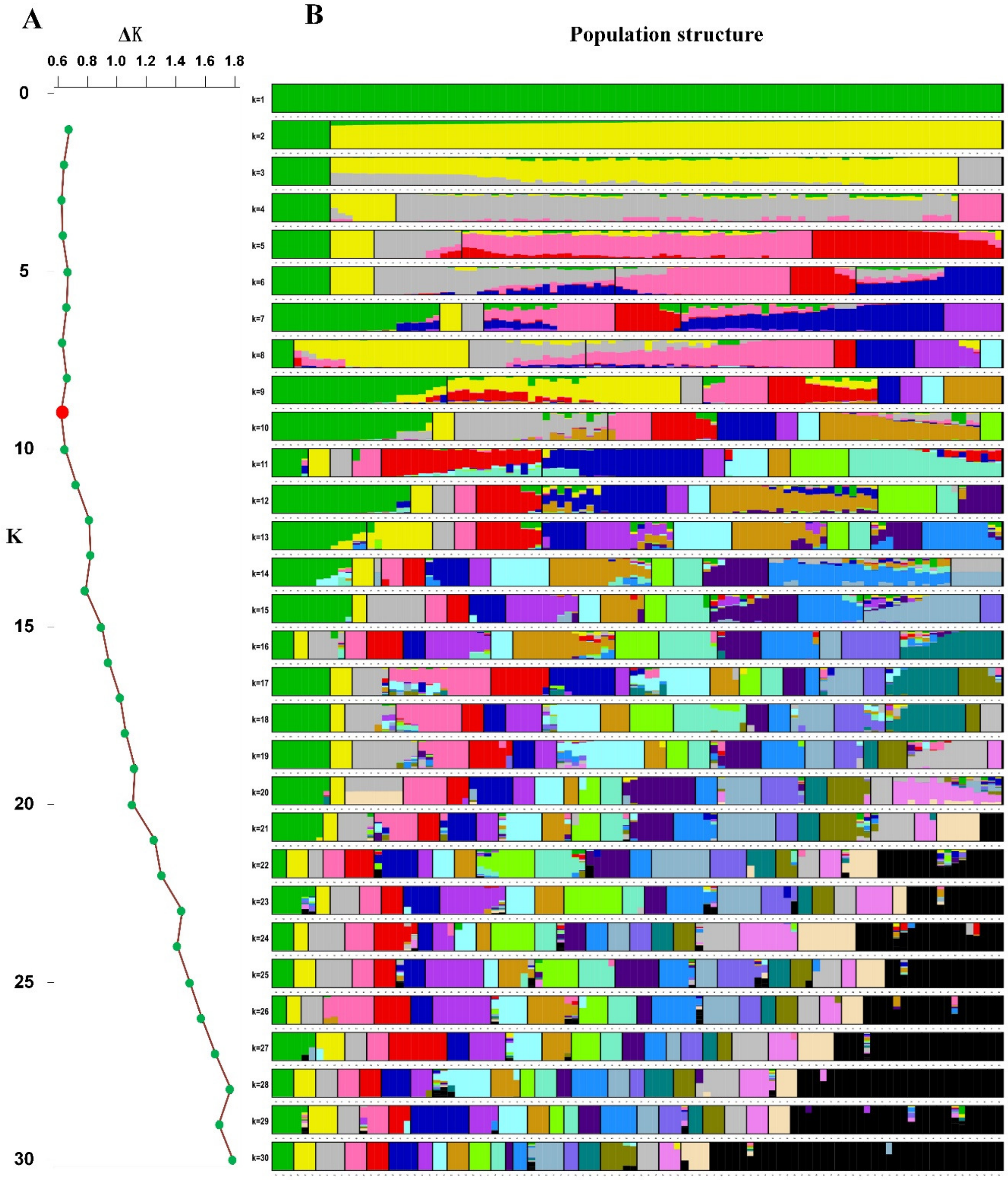

Supplementary Figure S3: Population structure of 100 accessions (**A**) and the individual clusters corresponding to each K value as determined with the Admixture software (**B**).

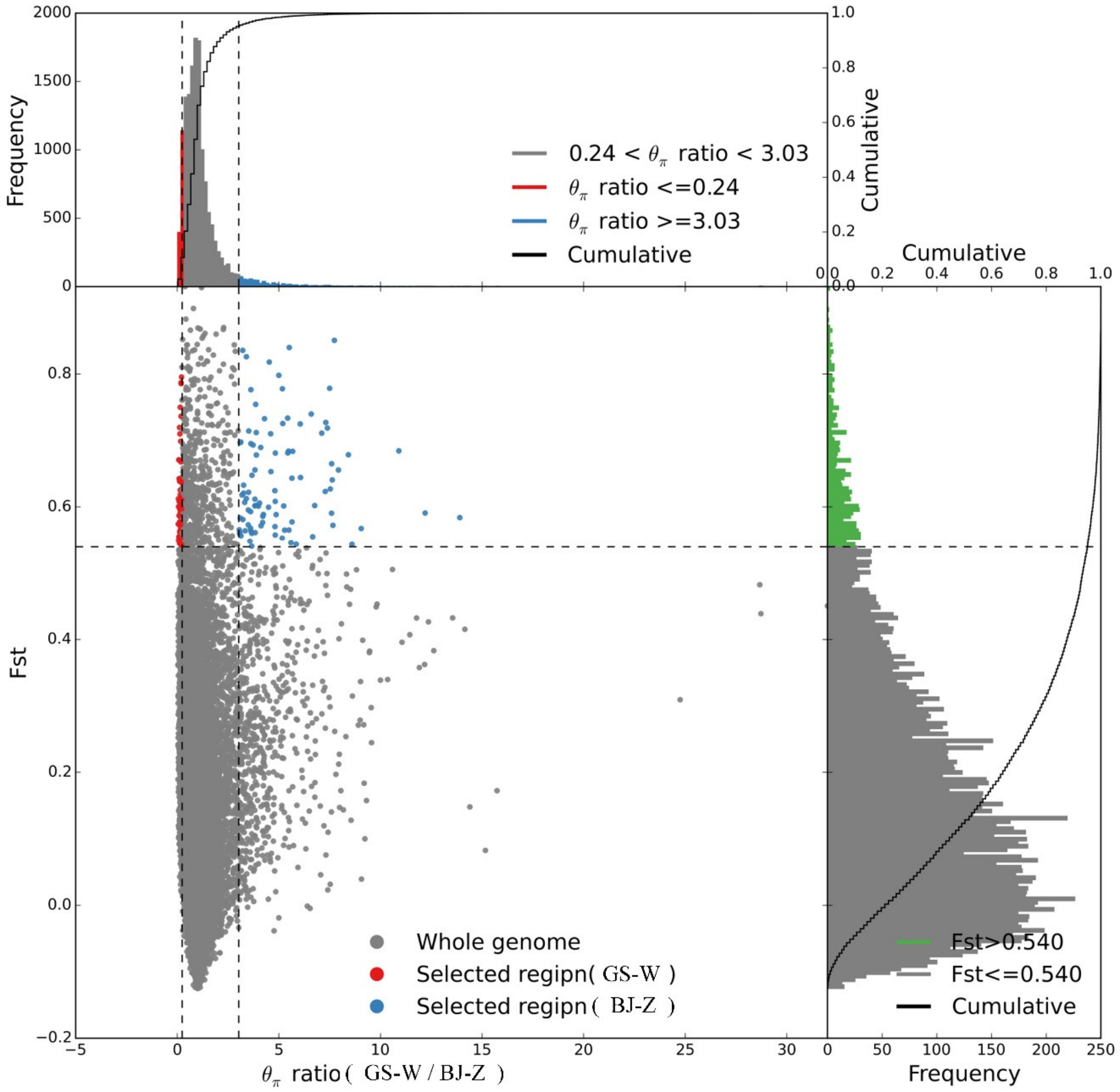

Supplementary Figure S4: Distribution of θπ ratios and Fst values and the regional distribution of population differentiation. Blue and green points represent the selected regions for the BJ-Z and GS-W populations.
